# Levelling the playing field for the international migration of nurses: the India English Language Programme

**DOI:** 10.1186/s12912-023-01308-7

**Published:** 2023-05-18

**Authors:** Ross Goldstone, Rose McCarthy, Ged Byrne, David Keen

**Affiliations:** 1 Global Health Partnerships, NHS England, 3 Piccadilly Place, Manchester, M1 3BN UK; 2 Global Health Partnerships, NHS England, Manchester, UK

**Keywords:** Education & training, Migration, Human resource, Workforce, Ethical, Global health

## Abstract

**Background:**

This article presents evaluation findings of the *India English Language Programme*, an innovative programme aimed at providing Indian nurses with an opportunity to participate in an ethical and mutually beneficial learning programme aimed at supporting migration into the United Kingdom’s National Health Service (NHS). The programme provided 249 Indian nurses wishing to migrate to the NHS on an ‘earn, learn, and return’ basis with funding to support English language learning and accreditation sufficient to apply for Nursing and Midwifery Council (NMC) registration. The Programme provided English language training and pastoral support to candidates, in addition to the availability of remedial training and examination entry for those not meeting NMC proficiency requirements on their first attempt.

**Methods:**

Descriptive statistical analysis of programme examination results and cost-effectiveness analysis is presented to demonstrate programme outputs and outcomes. Descriptive economic analysis of programme costings is presented alongside programme results to investigate the value-for-money provided by this programme.

**Results:**

A total of 89 nurses were successful in meeting NMC proficiency requirements, representing a pass rate of 40%. Those undertaking OET training and examination(s) were more successful, compared to those undertaking British Council provision, with over half of candidates passing at the required level. This equates to an overall programme cost-per-pass of £4139 and represents a model to support health worker migration, in line with WHO guidelines, delivering individual learning and development, mutual health system gain, and value-for-money.

**Conclusions:**

Taking place during the coronavirus pandemic, the programme evidences the effective delivery of online English language training to support health worker migration during a highly disruptive period for global health. This programme demonstrates an ethical and mutually beneficial pathway for English language improvement amongst internationally educated nurses to facilitate migration to and global health learning in the NHS. It provides a template through which healthcare leaders and nurse educators, working in policy and practice environments in the NHS and other English-speaking countries, can design future ethical health worker migration and training programmes to strengthen the global healthcare workforce.

## Introduction

Similar to most health systems globally, the National Health Service (NHS) continues to face workforce challenges [2, 3], with nursing shortages a key area of concern for the public, the workforce and those in management and leadership roles at service delivery level (National Audit Office, 2020 [9];). Mechanisms through which nursing workforce shortages can be addressed include both supply- and demand-side changes, including funding national education and training, investing and upskilling the current workforce, and international recruitment. International recruitment in the NHS continues to grow and there are increasing calls for a more ethical practice across the health system, specifically that which complies with the WHO Code of Conduct on International Recruitment of Health Professionals and is consistent with the Global strategy on human resources for health: Workforce 2030 (WHO, 2016) [14]. This Code of Practice was created in 2004 by the World Health Assembly in response to a growing concern regarding the growth in demand for health workers globally in a context of national workforce shortages in a multitude of countries, particularly but not exclusively high-income countries. The Code of Practice is underpinned by the principle of mutuality, meaning that international recruitment is permitted from any country via a mutually beneficial government-to-government agreement, even where a constituent country is among those deemed to have a critical shortage of health professionals [6]. In the UK, the Code of Practice was translated into regulations that stipulate the countries from which recruitment of health professionals is permitted in order to avoid health system workforce depletion and undermine efforts to achieve universal health coverage (UHC) [8, 15].

In this context, the United Kingdom (UK) Government commissioned Health Education England’s (HEE, now NHS England) Directorate of Global Health Partnerships to assist in increasing the number of nurses working in the NHS. This was one conduit through which the UK Government would meet its target of increasing the number of nurses working in the NHS by 50,000 by March 2024 [8]. To contribute to the number of nurses in the NHS in such a timeframe requires an ethical programme of international recruitment to be designed and initiated to supplement the continued training of those living in the UK aspiring to enter the profession.

Therefore, the India English Language Programme was initiated by HEE to support the ethical migration of nurses from India, an active supplier of health workers globally, to the NHS. The programme aimed to provide funding for English language training and examination to a cohort of Indian nurses who are required to meet Nursing and Midwifery Council (NMC) professional competence and English language proficiency requirements. These requirements were an overall IELTS score of 7, with minimum scores of 7 in reading, listening and speaking, and no less than 6.5 in writing or equivalent. International English Language Testing System (IELTS) test takers receive a score ranging from 1 to 9 in each of the four components, with the alternative examination type available being the Occupational English Test (OET) which grades candidates from E-A and numerically from 0 to 500. Both examination options are benchmarked against the Common European Framework of Reference for Languages (CEFR) (Table 1).

**Table 1:** IELTS and OET benchmarking

*CEFR Benchmark*	*IELTS*	*OET* *(incl. Numerical Score)*
C2	8.0–9.0	A (450–500)
C1	7.0–7.5	B (350–440)
B2	6.5	C+ (300–340)
	5.5–6.0	C (200–290)
B1	4.0–5.0	D (100–190)
A2	4.0	E (0–90)

The project focused on English language support because existing research shows that obtaining sufficient English language proficiency to meet UK health regulatory bodies’ standards is one of the largest issues obstructing health professionals, who are trained internationally, to migrate to the NHS or other English-speaking countries (Jamal et al., 2019 [10]). Although Hindi and English are the official languages of India, access to high-quality English language training opportunities is not universal and standards were found to vary substantially during a quality assurance assessment conducted in India during programme initiation.^1^ This means that English language proficiency and requirements stipulated by regulatory bodies, such as the NMC, remain a barrier to the migration of health professionals to the UK. Therefore, the programme would be of mutual benefit to the individual nurse through enabling professional and individual development via English language training and global learning opportunities [4], the NHS by increasing both the volume and diversity of the nursing workforce [1], and also the Indian health system and economy by providing employment and development opportunities to Indian nurses who (a) return with heightened skills associated with global learning and working and (b) are known to send remittance back to family whilst working abroad, which stimulates the local and national economy [11]. Moreover, those Indian nurses that are unsuccessful in meeting NMC English language proficiency requirements will still benefit from the English language upskilling enabled via education and training received.

Nurses who were successful in meeting NMC English language proficiency requirements would be permitted to progress onto the HEE Global Learners Programme (GLP). This was a programme offering overseas nurses with an opportunity to work in the NHS on an ‘earn, learn, and return’ basis. As part of the GLP, nurses would receive a suite of support to assist arrival in the United Kingdom, including full funding for flight, initial accommodation and visa costs, a structured induction and training programme at an NHS employer, and a full NHS salary for the duration of the programme. The aims of the intervention were as follows:

- Provide Indian nurses with an opportunity to participate in high-quality English language upskilling.
- Support nurses in meeting English language proficiency standards required by the NMC.
- Facilitate the ethical recruitment of highly skilled Indian nurses by NHS Trusts via supporting them to meet the English language requirements of the NMC.

Thus, the programme aims to contribute to levelling the playing field and expanding opportunity for active, economic migration for the international healthcare workforce, an activity which has traditionally been the preserve of health professionals from wealthier, developed countries [9]. In doing so, it offers an ethical conduit through which a ‘triple win’ can be achieved, seeing benefit for the NHS and service providers, the health professional, and the partner country (in this case, India).

The remainder of this article is as follows. Firstly, the India English Language Programme is outlined in further detail. Thereafter, information on the methodological approach taken in this programme evaluation and article is discussed. This is followed by a presentation of programme results, cost-effectiveness analysis and a discussion of the lessons that can be taken from this model for facilitating the migration of healthcare professionals more generally. Concluding remarks are then provided.

## The India English Language Programme

A total of 249 Indian nurses were enrolled onto the India English Language Programme, all of which had demonstrated English language proficiency at Common European Framework of Reference (CEFR) C1 level [5]. Nurses were recruited via a number of established partner organisations working in seven Indian states: Kerela, Tamil Nadu, Telangana, Andhra Pradesh, Punjab, and Delhi NCR. Partner organisations, most of which were aligned to local state governments, were required to pass a rigorous due diligence process where each organisation demonstrated:

- A track record in effectively working with the HEE Global Health Partnerships English language team during the Global Learners Programme
- Positive GLP candidate feedback
- Capacity to run English language training programmes
- Compliance with General Data Protection Regulation (GDPR); and
- A commitment to recruiting nurses directly and not subcontracting the recruitment of nurses to other suppliers

Each candidate was requested to make a bond payment totalling £50 upfront, which would be returned upon completion of the English language training and examinations. This was to maximise the personal investment made in the funded learning by each candidate and to reduce the withdrawal rate. In order to optimise programme success and value-for-money, and create an achievable offer for participants, programme entry was based on proficiency level. This decision was based on existing research on English language learning which demonstrates that, on average, 100 hours of face-to-face teaching and 100 hours of independent study is necessary to move from CEFR C1 to CEFR C2 [13]. Moreover, the total number of face-to-face teaching and independent study hours required for improvement increases as an individuals’ English language proficiency level descends the CEFR. Therefore, it was imperative to ensure nurses enrolled onto the programme with a sufficient level of English language proficiency to enable progression to NMC requirements. Prospective candidates were able to demonstrate sufficient English language proficiency by either providing examination transcripts for a recognised English language examination undertaken in the previous 2 years (Route A) or undertaking a British Council or Occupational English Test (OET) baseline screening test (Route B). Most (82%) enrolled candidates were Route A candidates.

Each enrolled candidate undertook a course of English language study delivered by British Council or Occupational English Test (OET) providers in India based on the candidate’s preferred English language examination. British and OET were chosen in this programme because they were the only providers able to quality assure teaching and examination support. Almost two-thirds (65%) of candidates chose OET provision, with the remaining 35% opting for British Council examinations. In response to the coronavirus pandemic and national restrictions imposed across India during the programme, all learning and most English language examinations were delivered online. A summary of the British Council and OET provision, both of which lasted for 12-weeks, is provided in Table 2.

**Table 2:** English language provision summary

British Council	OET Lite & FMS	OET FMS
One-hour orientation	Minimum learning time of 200 hours, inclusive of self-study, homework, and mock examinations	Work with suppliers to support marketing and recruitment to the courses.
IELTS Coach lessons & App, comprising 30 hours of both taught and self-directed reading and listening practice	Performance of a needs analysis by the provider with assistance from OET	Manage all aspects of communication to the candidates on course details & queries from candidates.
Road to IELTS, where participants were able to access authentic test materials and test strategies (access provided for a year). Textual analysis/peer correction tasks, providing seven-and-a-half hours of weekly tasks focusing on the language and style necessary to achieve the required 6.5 examination score	Creation of individual candidate learning plans by the EL providers	Development of a sense of network and collegial support between candidates. Provide wrap-around support for candidates ensuring excellent communication and guidance is in place, dealing with any issues which are raised by candidates and ensuring that candidates have a smooth journey through the programme.
Weekly writing tasks, enabling students to engage in 15-hours of self-directed writing activities with individual feedback provided by an EL trainer.	Classes to have a minimum 80% focus on skills and 20% mock examination time	Work with the provider to manage candidates’ journeys, track attendance & engagement with the provider &, where appropriate, communicate with candidates to encourage engagement in the course or identify issues that they may have.
One-to-one mid-course meetings with EL trainers	Four teacher feedback sessions for candidates	Work with candidates to ensure they know their exam date & encourage attendance.
Tutor Group, consisting of five hours spent in collaborative learning groups	Relearn opportunity, where additional learning, coaching, examination preparation and pastoral support was delivered before a second examination	

OET candidates were offered a place on one of two packages: (1) the Fully Managed Service (FMS) and (2) the Lite service. Allocation to each package depended on region and availability. Both packages were comprised of core specifications outlined in the second column of Table 2, but the FMS contained additional support services from OET to HEE, which are outlined in the third column. OET candidates who failed to meet NMC English language proficiency requirements in the post-course examination had the opportunity to undertake relearn provision. This delivered additional learning and coaching, examination preparation and pastoral support to candidates who wished to undertake a second attempt to meet NMC English language requirements.

All teaching and learning were based on a Quality Assurance Framework, which was created in conjunction with language specialists at the University of Salford, England. The programme operated from November 2020 to September 2021, with all English language training, including relearn opportunities, completed by May 2021. All examinations were completed by September 2021.

Indian nurses who were successful in meeting NMC English language proficiency requirements would be permitted to progress onto the HEE GLP. This aimed to facilitate the appointment of international nurses seeking educational and employment opportunities in participating English NHS trusts. The programme would support candidates and NHS trusts at all stages of the recruitment process, including application submission, screening and shortlisting, as well as interviewing, issuing of employment offers and conducting pre-employment checks. Once appointed by an English NHS trust, a four stage support package was available to GLP recruited nurses:

1. Preparation and support prior to coming to England, including Computer Based Test (CBT) preparation, NHS introductory learning materials, and support transitioning to UK living.
2. Orientation, preparation and support to gain NMC registration after arrival in England.
3. Post-NMC registration support and development, including enrolment on a preceptorship programme and engagement in other local professional development opportunities.
4. Continued support mechanisms, such as educational opportunities at each respective organisation, mentoring and buddy schemes.

Where candidates were unsuccessful in meeting the NMC English language proficiency requirements in the funded English language examinations, they were unfortunately unable to be supported further by this programme. It is for this reason that entry onto the programme required a high level of starting proficiency in order to minimise examination failure.

## Method

An ex-post evaluation of the India English Language Programme was conducted following programme completion.

### Methods

Evaluation data was collected from English language providers on individual candidates’ progression and programme outcomes, of which the variables used in this article are:

- Candidate enrolment
- Candidate programme withdrawal
- Candidate English language course completion
- Candidate post-course examination result
- Candidate destination data

### Analysis

Descriptive data analysis of programme statistical data at programme completion was conducted using Microsoft Excel to investigate the number of course completions, examination completions, examination passes at the NMC English language proficiency requirements, and course pass rates according to enrolment route and English language provision type (i.e., British Council or OET). In order to assess the added value of the relearn opportunity provided by OET providers, data is also presented of programme pass rates for relearn candidates and overall programme pass rates. In addition, an analysis was conducted of financial costings relative to pass rates for each English language provision type. This financial analysis enables an assessment of (a) the relative value-for-money of each provider’s service and (b) the cost of facilitating the migration of international nurses into the NHS via the model adopted in this programme compared to other forms of nurse recruitment.

### Participants

Data was obtained and analysed for all 249 participants on the India English Language Programme. Demographic data is unavailable for programme participants in line with a data sharing agreement agreed during the programme. Inclusion criteria for this study was that the nurses must have possessed a minimum English language proficiency level of CEFR B2 (IELTS 6.5 or equivalent).

### Ethics

In line with the internal ethical review process at Health Education England, programme candidates provided consent for programme data to be used for evaluation and research purposes. Consent was gained via completion of a data protection agreement signed by the candidate at enrolment stage. No personal or identifiable data is presented in any output included in this publication and this research did not involve animal subjects. All data collected was stored on encrypted Health Education England (HEE) servers and stored in password-protected files to ensure data security.

## Results

Of the 249 enrolled candidates enrolled onto the India English Language Programme, a total of 26 withdrew either before starting provision or promptly after beginning English language training. These candidates are excluded from analysis on programme pass rates provided in Tables 3, 4 and 5 yet are included in financial assessment data presented in Table 6 as costs were incurred during enrolment.

**Table 3:** Post-course examination results by route and type of English language provision

		Tests (N)	Passes (N)	Pass Rate (%)
Route	Route A	179	34	19
	Route B	44	11	25
Partner	OET	150	33	22
	British Council	73	12	16
Overall		**223**	**46**	**21**

**Table 4:** Relearn course test results by route and type of English language provision

		Tests (N)	Passes (N)	Pass Rate (%)
Route	Route A	65	35	54
	Route B	15	8	53
Partner	OET	73	43	59
	British Council	7	0	0
Overall		80	43	54

**Table 5:** Overall programme results, inclusive of post-course and relearn results

		Tests (N)	Passes (N)	Pass Rate (%)
Route	Route A	178	70	39
	Route B	45	19	42
Partner	OET	150	77	51
	British Council	73	12	16
Overall		223	89	40

**Table 6:** Programme costings by type of English language provision

	Total Costs	Enrolled Candidates	Cost Per Candidate	Completed Exams	Cost Per Exam	Passing Candidates	Cost Per Pass
British Council	97239	87	1118	73	1332	12	8103
OET	271145	162	1674	150	1808	77	3521
Total	368384	249	1485	223	1652	89	4139

Table 3 displays the number of tests and passes at the NMC English language proficiency requirement and pass rates by programme entry route and type of English language provider. Among the 223 candidates completing English language training and the post-course examination, an overall pass rate of 21% was achieved. Pass rates were highest among candidates entering the programme following completion of a baseline screening test (Route B) compared to those entering via Route A (25% compared to 19%). Likewise, candidates completing OET English language training and undertaking the OET examination achieved a higher pass rate than British Council candidates (22% compared to 16%).

As previously outlined, OET English language providers delivered remedial relearn opportunities for many nurses who were unsuccessful in meeting NMC English language proficiency requirements in the post-course examination. Table 4 shows that the majority of nurses completing the relearn opportunity were Route A candidates (81%) and OET candidates (91%). Pass rates by route of entry did not differ substantially, with a single percentage point difference between Route A (54%) and Route B (53%) pass rates.

The majority (59%) of OET candidates undertaking the relearn opportunity successfully met NMC English language proficiency requirements. Only seven British Council candidates pursude the OET relearn opportunity, all of which were unsuccessful in meeting NMC English language proficiency requirements.

Table 5 combines post-course and relearn examination data to present overall programme results. This shows an overall programme pass rate of 40%. Differences in pass rates are negligible when comparing the route of entry into the programme. Yet, a large difference is observed in programme pass rates between OET and British Council candidates. Post-course examination results showed a 6-percentage point difference between type of English language provision (22% compared to 16%) (Table 3). However, following the inclusion of OET relearn examination results the differential increases to 35-percentage points (51% compared to 16%). This data suggests that OET English language provision provided as part of the India English Language Programme is a more effective delivery method for improving the English language proficiency for Indian nurses interested in migrating to the United Kingdom to work in the NHS, especially following the provision of remedial relearn learning opportunities to candidates.

**Fig. 1:**
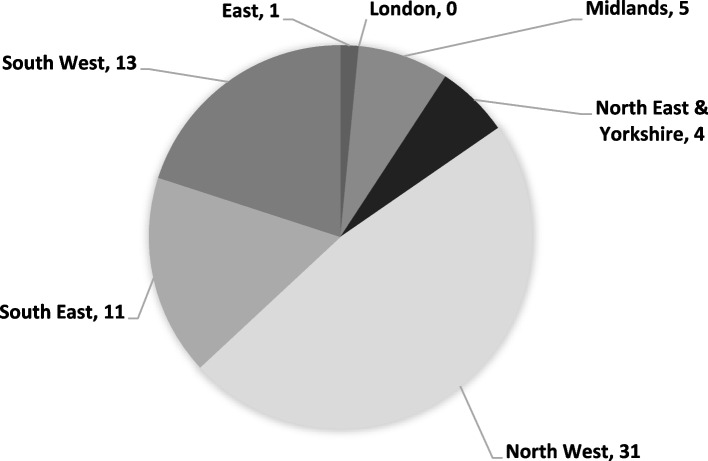
Destination Data for India EL Programme GLP candidates by NHS Region. Sample: 65. Note: The status is currently unknown for six candidates

Of the 89 successful programme candidates, 71 (80%) opted to pursue NHS employment via the GLP, which offered the Indian nurses an opportunity to work in the NHS on an ‘earn, learn, and return’ basis. Figure 1 displays destination data for these candidates by NHS region.

Table 6 combines programme results data with programme financial data to enable an assessment of which form of provision offered the highest value-for-money for HEE during this programme. Overall, this demonstrates that £4139 per Indian nurse passing at the NMC English language proficiency requirement was achieved in this programme. Table 5 stratifies programme costings by type of English language provider used. This shows that OET offered the strongest returns on investment and highest value-for-money by achieving £3521 per passing candidate, as compared to British Council provision which achieved a cost per pass of £8103.

## Discussion

Findings presented in this article demonstrate that OET English language provision and examinations produced stronger results, relative to those candidates undertaking British Council provision, among Indian nurses wishing to migrate to the NHS. Stronger results are achieved both in terms of (a) overall pass rates, with more than half of OET candidates meeting NMC English language proficiency requirements, and (b) value-for-money, where the OET achieved a cost-per-pass more than half the value of British Council candidates. However, British Council did not provide a relearn opportunity for candidates, resulting in only seven British Council candidates pursuing the relearn opportunity, all of which were unsuccessful in meeting NMC English language proficiency requirements. This may be one factor for the differential pass rate achieved. This is for two reasons. Firstly, because OET candidates received further tuition following their first examination attempt (if unsuccessful). Secondly, among the seven who did undertake the relearn course, the content was designed for the OET examination, thus limiting its applicability for IELTS preparation. Furthermore, the difference between British Council and OET examinations may be partially responsible for these findings. OET is an English language test designed specifically for healthcare professionals, which assesses the language and communication skills of international healthcare workers who wish to enter clinical practice in an English-speaking country. On the other hand, the International English Language Testing System (IELTS) examination is a general academic English language examination which examines a candidates’ listening, reading, writing, and speaking skills. Whilst OET is designed principally to facilitate healthcare professional migration to English-speaking countries, IELTS is not specially designed for health professionals and is the standard examination used by prospective students from non-English speaking countries aspiring to enter a Higher Education Institution in the United Kingdom. Thus, the greater vocational relevance of the OET examination may assist in the higher pass rates achieved by Indian nurses participating in this programme. However, the enhanced English language training and support provided via the OET delivery model, including the Relearn opportunity, may have also influenced the pass rate achieved. Also, the number of passes almost doubled (from 46 to 89 nurses) following relearn course and examination completion, most of which were among OET candidates. This finding underlies the importance of high-quality English language training which is comprised of remedial support for those candidates that are unsuccessful on their first attempt.

The results of this programme also suggest that prior demonstration of English language proficiency is not an indicator of higher proficiency level and probability of meeting NMC English language requirements. On the contrary, enrolled candidates entering the programme via Route B had a slightly higher pass rates overall than Route A counterparts (42% compared to 39%). This finding, although requiring future research to explore further, suggests that baseline testing is significantly important in programmes aimed at improving English language proficiency among health professionals and ensuring that those most able to benefit from such education and training are supported.

Given the increasing recognition that the NHS workforce is recognised as both its most valuable asset and in need of further investment, these findings are of contemporary significance to the NHS. The programme illustrates how workforce challenges faced by nurse management can be addressed through a combination of high-quality education and training and international recruitment facilitated via education. Ethical International recruitment is shown in this programme to be able to deliver value-for-money and mutual benefit to the NHS and its service providers, the health professional, and the partner country. Benefit for the NHS is achieved through investment in its long-term workforce needs, contributing further to the diversity of the NHS workforce, and to the Indian diaspora working in the NHS and representing the second largest nationality among NHS employees [7, 12]. For health professionals, these programmes offer access to learning and development opportunities in the NHS, access to which is known to be inequitable and necessitates an uncertain and expensive journey with no guarantee of success. Through creating more equitable migration pathways for health professionals, ethical recruitment programmes can offer benefits to origin country health systems too. This is demonstrated in this programme through supporting the Indian Government in creating migratory opportunities to highly trained health professionals who would otherwise have no employment in healthcare in India, which is supported by Indian government agencies like Overseas Development and Employment Promotion Consultants (ODEPC) [16]. Furthermore, whilst the programme might be perceived to involve the depletion of local Indian health systems of highly trained nursing professionals, HEE did work in partnership with state governments in India when establishing this programme. Hence, this programme is consistent with the principle of mutuality underpinning the WHO Code of Practice, yet we actively appreciate the tension implicit in work of this kind.

Ultimately, what this programme indicates is the global asset the NHS represents for the UK and how it is a conduit through which the UK can contribute to both its national challenges, its global responsibilities and forming health partnerships with partner health systems.

This article does possess some limitations. Firstly, it should be noted that this programme was conducted in a single country – India – which legally stipulates English as one of its official languages. This raises a potential limitation on the generalisability of the findings of the results presented, but moreover demonstrates the difficulty experienced by international nurses in meeting the English language proficiency requirements of the NMC. Secondly, entry onto the India English Language Programme was only available for Indian nurses with a high level of prior English language proficiency. Therefore, it is unclear how representative the results of this project are to those Indian nurses who have lower levels of proficiency. Thirdly, the absence of demographic and multivariate analyses associated with such data limits the explanatory power of conclusions made. Fourthly, results only focus on direct and immediate results and costings from the project, ignoring the indirect and long-term impacts of participation in the programme and migration for the individual, NHS system and Indian health system. This inhibits a complete analysis of the mutually beneficial nature of this programme and the trajectories followed by participants. Furthermore, this is compounded by the incomplete responses on the current status of programme candidates and GLP entrants (see Fig. 1). Further research and evaluation should be conducted on similar programmes of work to supplement the findings of this article.

## Conclusion

To conclude, the India English Language Programme was designed to facilitate the ethical migration of Indian nurses to the UK to work in the NHS. It uniquely provided a structured and funded educational opportunity to Indian nurses to migrate and engage in global health learning and development in the NHS. In doing so, it aimed to achieve a ‘triple-win’ by: (a) contributing to increasing the NHS nursing workforce, (b) providing Indian nurses with an opportunity to work in the NHS and develop an array of skills associated with working in other health systems, (c) which will enable them to contribute to the Indian health system in the future. It is hoped that this model of healthcare professional migration, which placed principals of working ethically and mutual benefit at the heart of its design, will be of value to those involved in managing, educating and supporting the nurse workforce, particularly nursing management, in English-speaking countries that are currently facing and are projected to continue to encounter workforce shortages in the years to come. However, it also provides a template to improve the design and enhance the ethical nature of recruitment activity in countries across the world.

### Implications for nursing management

This article provides exemplifies how nursing management in the NHS and other English-speaking countries can design ethical recruitment practices in collaboration with partner countries for mutual benefit. The results also suggest important factors that nursing management in the NHS and other anglophone health system should consider when providing English language upskilling to support recruitment of international nurses. This is of heightened importance given recent developments in NMC English language standards and the interest NHS service providers has shown in proposed changes.

## Data Availability

The datasets generated and/or analysed during the current study are not publicly available due regulations governing the use of personal data but are available from the corresponding author on reasonable request.

## Abbreviations

CEFR: Common European Framework of Reference
CBT: Computer Based Test
GDPR: General Data Protection Regulation
HEE: Health Education England
IELTS: International English Language Testing System
GLP: Global Learners Programme
NHS: National Health Service
NMC: Nursing & Midwifery Council
OET: Occupational English Test
UHC: Universal Health Coverage
WHO: World Health Organization

## Footnotes

1 During the initiation phase of the India English Language Programme, Health Education England conducted an in-country assessment of English language provision in January 2020, with members of the assessment panel including nursing professionals and an English language academic from a UK higher education institution (HEI). Quality assurance assessments were conducted at eight English language providers in three Indian states adopting a mixed-methods approach, including observations, interviews, focus groups and document analysis.
